# Sacral Agenesis Type II in a 12-Year-Old Patient: A Delayed Presentation in a Low-Resource Setting

**DOI:** 10.7759/cureus.86903

**Published:** 2025-06-28

**Authors:** Muhammad Ayub, Quang Dai La, Aiman Baloch, Sobia Ahmed

**Affiliations:** 1 Radiology, Bolan Medical Complex Hospital, Quetta, PAK; 2 Surgery, The Innovative STEMagazine, College Station, USA; 3 Medicine, Mekran Medical College, Turbat, Turbat, PAK; 4 Medicine, The Innovative STEMagazine, College Station, USA

**Keywords:** caudal regression syndrome, coccyx, sacral agenesis, sacrum, socio-economic factors

## Abstract

Sacral agenesis is a rare congenital anomaly with varying severity, from partial sacral hypoplasia to complete absence of the sacrum and coccyx. Often linked to maternal diabetes, it can cause significant neuromuscular and genitourinary complications. We present a 12-year-old female patient from a low-resource setting with lifelong lower limb weakness and urinary incontinence, born to a diabetic mother and diagnosed with sacral agenesis Type II. Financial constraints limited further management. This case highlights the challenges of delayed diagnosis and treatment, underscoring the need for improved healthcare access to ensure timely intervention for congenital disorders, particularly in underserved populations.

## Introduction

Sacral agenesis, also known as caudal regression syndrome, is a rare congenital anomaly characterized by the incomplete development of the lower spine and sacrum [1]. This condition presents with a variable spectrum of severity, ranging from mild sacral hypoplasia to complete absence of the sacrum and coccyx, often accompanied by associated anomalies affecting the genitourinary, gastrointestinal, and musculoskeletal systems [1]. The different severities of this condition are classified into various types, using Renshaw's classification [2]. Although sacral agenesis is already a rare occurrence, the presentation of this syndrome at an early age is increasingly rare.

The exact etiology remains unclear, although several factors have been implicated, including genetic predisposition, teratogenic influences, and, most notably, maternal diabetes mellitus [3]. Maternal diabetes, particularly poorly controlled gestational diabetes, significantly increases the risk of sacral agenesis, with a reported relative risk of a 200-fold increase in affected infants [4]. However, a significant number of cases occur in non-diabetic mothers, highlighting the complex interplay of genetic and environmental factors in the pathogenesis of this condition [5].

The worldwide incidence of sacral agenesis has been estimated to be about 0.01-0.05 cases per 1,000 live births, although there is still regional variation, and epidemiological data from low-resource settings are limited. The clinical presentation of sacral agenesis ranges from simple, isolated neurologic deficits to more complex, multisystem involvement, and the infectious diagnosis is significantly related to the phenotypic severity of sacral agenesis, as well as the syndromic conditions associated with it [6]. In low-resource settings, diagnosis can be difficult because prenatal imaging and MRIs are limited, which can result in delayed diagnosis and intervention. Treatment typically involves a multidisciplinary approach that incorporates specialists from orthopedics, neurology, urology, and rehabilitation, which can be a challenge to achieve in low-resource regions [1].

Many published case reports detail the diverse mechanisms and phenotypes of the disorder, ranging from isolated neurologic deficits to complex multisystem involvement, and also highlight the importance of individualized management and long-term follow-up, particularly concerning function and quality of life [1].

## Case presentation

A 12-year-old female, born to a diabetic mother, presented to the outpatient clinic with complaints of bilateral lower limb weakness and dribbling of urine since birth. This marked the patient's first contact with a healthcare facility, as her family, belonging to a low socioeconomic group, had previously neglected her condition.

**Figure 1:**
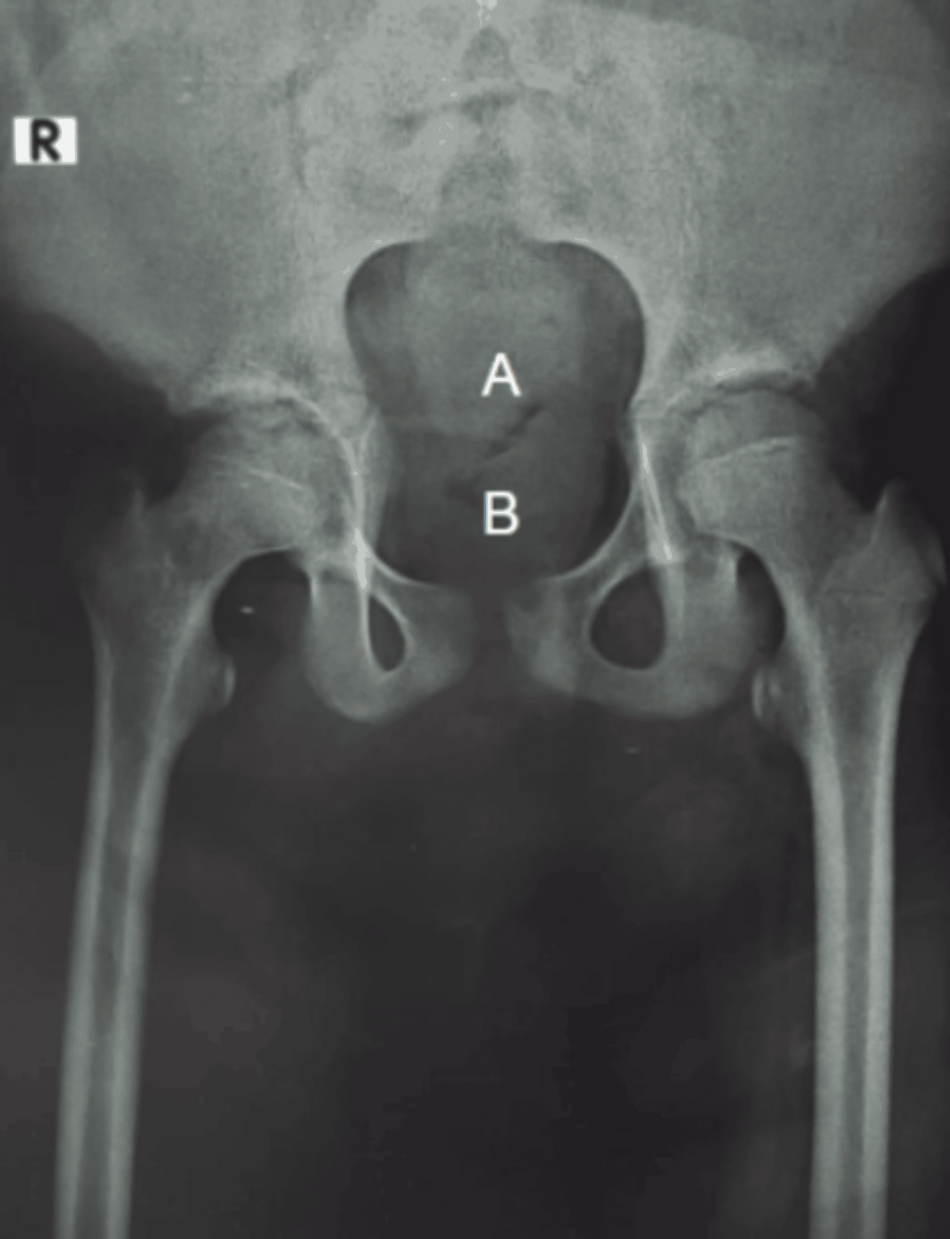
Frontal radiograph of the pelvis showing non-visualization of the (A) sacrum and (B) coccyx.

On physical examination, the patient exhibited slightly hypoplastic lower limb muscles and narrow hips. The initial evaluation included a radiograph, which revealed the non-visualization of the sacrum and coccyx (Figure 1).

**Figure 2:**
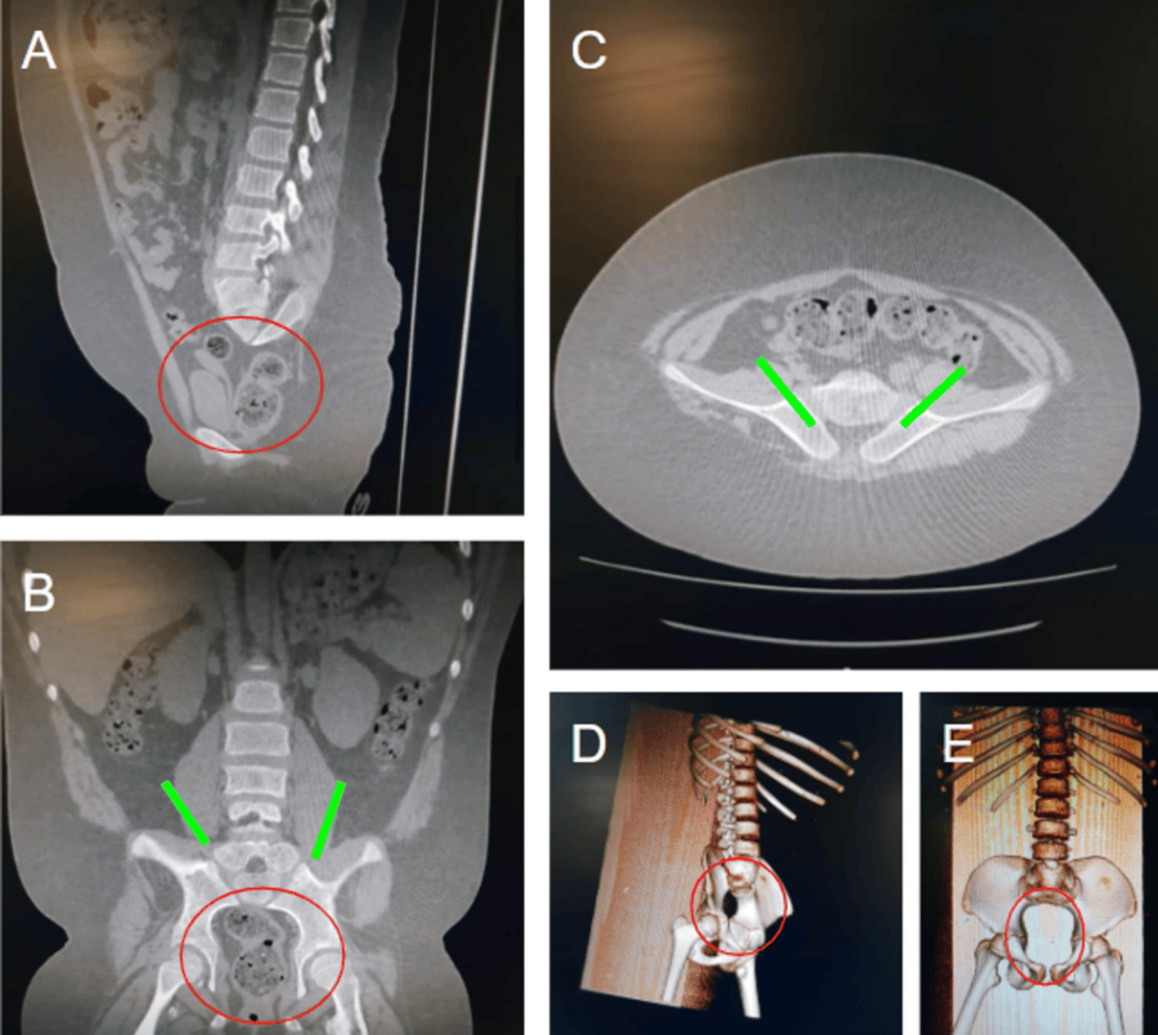
(A) Sagittal, (B) coronal, (C) axial, and (D,E) 3D reconstructed CT images showing bilateral iliac bones articulating with the S1 vertebra with reduced distance between them. There is an absence of the rest of the sacral vertebrae and coccyx. Green lines: Bilateral iliac bones articulating with the S1 vertebra, with reduced distance between them. Red circles: Absence of the rest of the sacral vertebrae and coccyx.

Further assessment was conducted using three-dimensional imaging studies. These studies demonstrated the visualization of the first sacral vertebra (S1) with a complete absence of the remaining sacrum and coccyx. There was a decreased distance between the bilateral iliac blades, which were articulating with the S1 vertebra (Figure 2).

**Figure 3:**
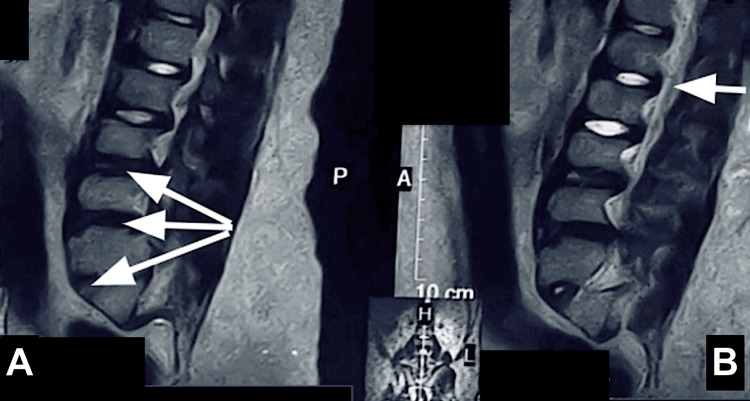
(A) Sagittal T2WI showing disc desiccatory changes at the L3-L4, L4-L5, and L5-S1 levels with reduced disc height at the L5-S1 level. (B) Sagittal T2WI showing a hypoplastic spinal cord. T2WIs: T2-weighted images

The spinal cord appeared hypoplastic and poorly visualized, with the conus medullaris terminating at the level of D12-L1. Sagittal T2-weighted images further demonstrated a hypoplastic spinal cord with disc desiccatory changes at the L3-L4 and L5-S1 levels, along with reduced intervertebral disc height at L5-S1 (Figure 3). No evidence of defects in the posterior elements of the visualized spine or any associated meningocele or myelomeningocele was observed. Additionally, partial disc dehydration and reduced intervertebral disc height were noted at the level of L5-S1.

Based on the patient's history, clinical examination, and radiological findings, a diagnosis of sacral agenesis (Type II) was made, and the patient was referred to a multidisciplinary team for further management. The patient's family rejected further examination due to economic restrictions.

## Discussion

Sacral agenesis is a rare congenital malformation disorder characterized by incomplete development of the sacrum, often accompanied by malformations in the musculoskeletal, genitourinary, and gastrointestinal systems [7]. The current case demonstrates the challenges of identifying and managing sacral agenesis in an under-resourced setting, where outcomes for all patients are negatively impacted by delays in presentation and limited patient resources. The case contributes to the medical knowledge base, focusing on the importance of early identification and management whenever possible, particularly within disadvantaged populations that already face limitations in accessing specialist treatment. Additionally, a limitation of this case is the lack of genetic testing, which may further validate the findings. Sacral agenesis is typically due to the disruption of caudal embryologic development of the notochord and mesoderm, typically occurring between the fourth and seventh week of gestation. Sacral agenesis is classified into four types, including Type II, where there is incomplete sacral agenesis and the iliac bones articulate with the lowest present vertebra [8]. Maternal diabetes has been singled out as an important risk factor for sacral agenesis, but other proposed causes include genetic mutations, teratogenic exposure, or vascular disruptions in the caudal region [9]. In the present case, the patient was born to a diabetic mother, supporting the hypothesis that maternal hyperglycemia is a significant contributor to abnormal fetal development.

With an estimated prevalence of one in 25,000 live births, sacral agenesis is a rare disorder [10]. Clinically, it presents with a wide range of symptoms, from minor musculoskeletal abnormalities to severe neurological impairments [11]. According to earlier findings on sacral agenesis, the patient in this instance had lower limb paralysis and urine incontinence [12]. Significant functional deficits resulted from the lack of early medical intervention, underscoring the consequences of postponed diagnosis and restricted access to specialized care [13,14].

Sacral agenesis can be mistaken for several illnesses, so a careful differential diagnosis is required [15]. Although myelomeningocele and lipomyelomeningocele, two types of spinal dysraphism, have comparable neurological impairments, they are frequently linked to midline cutaneous indicators [16]. Advanced imaging modalities are necessary to effectively identify other differential diagnoses, such as vertebral segmentation abnormalities and tethered cord syndrome. Other congenital spinal abnormalities were ruled out in this instance after the diagnosis was verified by radiography and three-dimensional imaging [17,18].

Orthopedic, neurological, urological, and rehabilitation professionals are involved in the interdisciplinary care of sacral agenesis [13,19]. Mobility, continence, and general quality of life can all be enhanced with early intervention. For neurogenic bladder dysfunction, treatment options include orthopedic adjustments, bladder augmentation, and surgical treatments such as spinal stabilization [20]. However, because of financial limitations, a typical obstacle in low-resource environments, the patient's family declined additional therapy.

If diagnosed early, timely interventions such as bracing, neuromuscular rehabilitation, bladder management strategies, and orthopedic corrections could have greatly reduced functional impairments and improved eventual prognosis [21].

In such settings, conservative management approaches, such as physical therapy to improve lower limb strength, the use of orthotic devices for mobility support, and clean intermittent catheterization for managing a neurogenic bladder, may help maintain function and prevent secondary complications. This highlights the urgent need for initiatives that provide access to healthcare and financial assistance for affected individuals [22].

## Conclusions

This case illustrates the profound impact of sacral agenesis on an individual's functional status, particularly when diagnosis and treatment are delayed due to socioeconomic limitations. While medical advancements offer promising treatment options, their availability remains a challenge in low-resource regions. Increased awareness, early screening programs, and improved healthcare infrastructure are essential to addressing these disparities and improving outcomes for patients with congenital spinal anomalies.
